# Particular dental erosion

**DOI:** 10.11604/pamj.2018.30.190.16196

**Published:** 2018-07-02

**Authors:** Giovanna Mosaico, Cinzia Casu

**Affiliations:** 1 DDS, Private Dental Practice, Cagliari, Italy

**Keywords:** Lemon juice lesion, dental erosion, enamel pigmentation

## Image in medicine

A 30-year-old smoker female patient in good systemic health came to our attention for the professional oral hygiene session. On clinical observation, the patient had a severe erosion of the enamel, dentinal hypersensitivity and excessive pigmentation of eroded areas. These lesions on the enamel, with consequent exposure of the dentin, was caused by exogenous acids: in fact the patient reported having taken lemon juice for about a year during main meals and as a snack. Every morning, for the whole period indicated, she had taken water and lemon juice; at lunch and dinner and during snacks, based exclusively on fruit. She reported having made use of lemon and vinegar juice on salads and fruit, and finally she drank the lemon juice with great frequency after meals. Rubbing the teeth with more force, in the attempt to remove the pigmentations a chain mechanism has been triggered between acidic demineralization and mechanical abrasion of the enamel with increasingly evident dentine exposure. The patient will be subjected to remineralizing agents and to a conservative prosthetic rehabilitation. The differential diagnosis arises with damage from chemical trauma produced by other fruits such as orange juice and lime; alterations of salivary pH, excessive use of bleaching agents. The anamnesis was decisive in this case.

**Figure 1:**
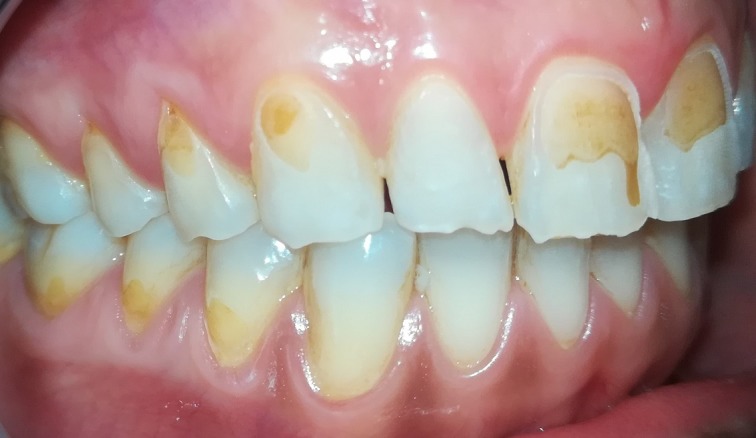
Enamel and dentin erosion caused by lemon juice

